# Supplementary material for: Synthesis and Characterization of Newly Designed and Highly Solvatochromic Double Squaraine Dye for Sensitive and Selective Recognition towards Cu2+
Source: Molecules. 2022 Oct 4;27(19):6578. doi: 10.3390/molecules27196578 (PMC9571602; doi:10.3390/molecules27196578)
Supplement: Supplementary file 1 [file molecules-27-06578-s001.zip › molecules-1938890-supplementary.pdf]

Supplementary Material

Synthesis and Characterization of Newly Designed and Highly Solvatochromic Double Squaraine Dye for Sensitive and Selective Recognition towards Cu^{2+}

Linjun Tang*, Shubham Sharma and Shyam S. Pandey*

1. NMR spectral analysis:

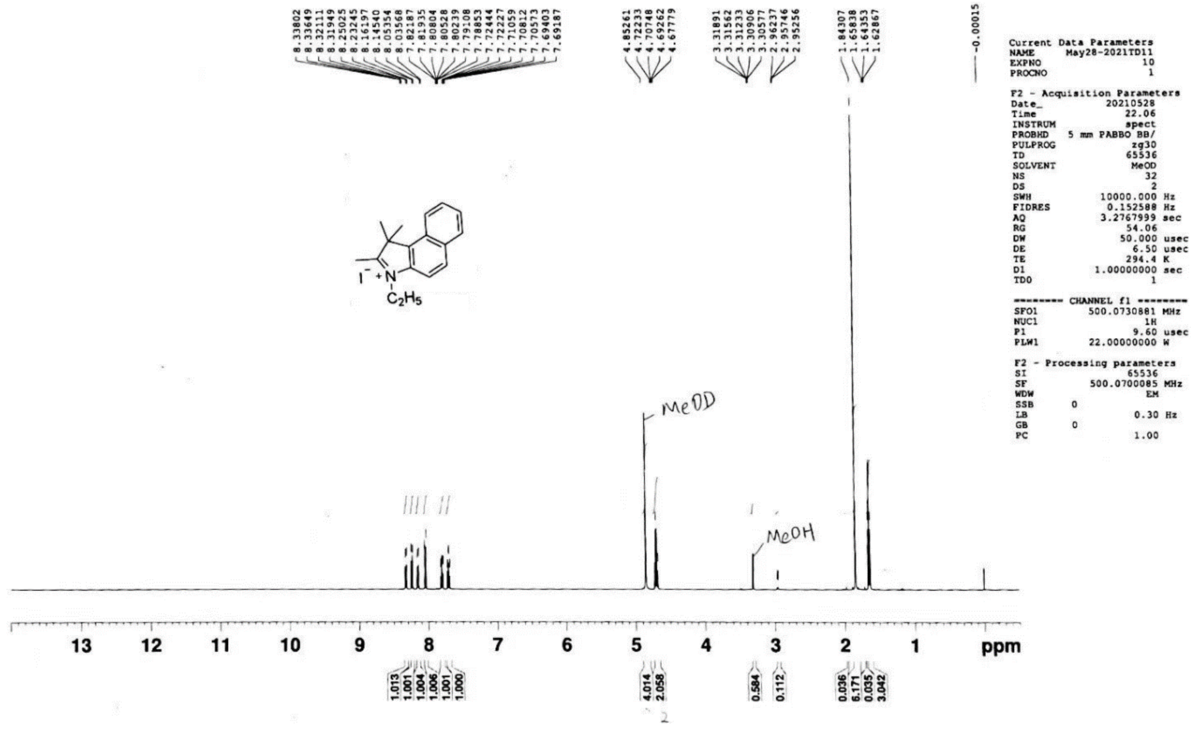

Figure S1. ^{1}H-NMR spectrum of **3** in MeOD.

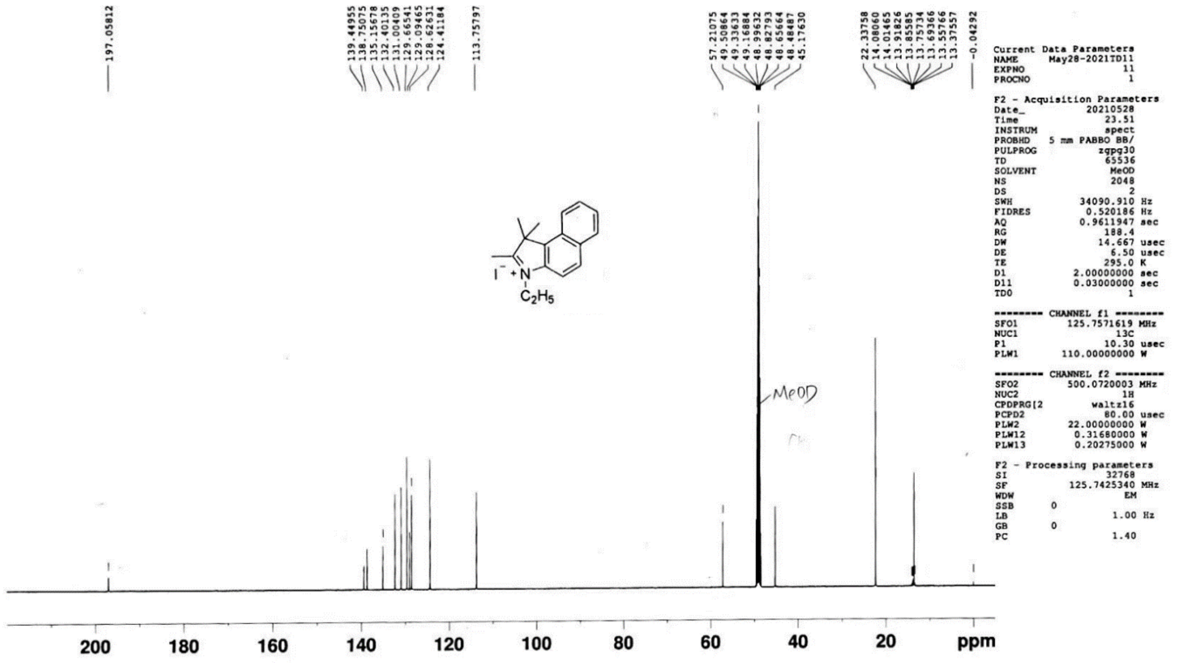

Figure S2. ^{13}C-NMR spectrum of **3** in MeOD.

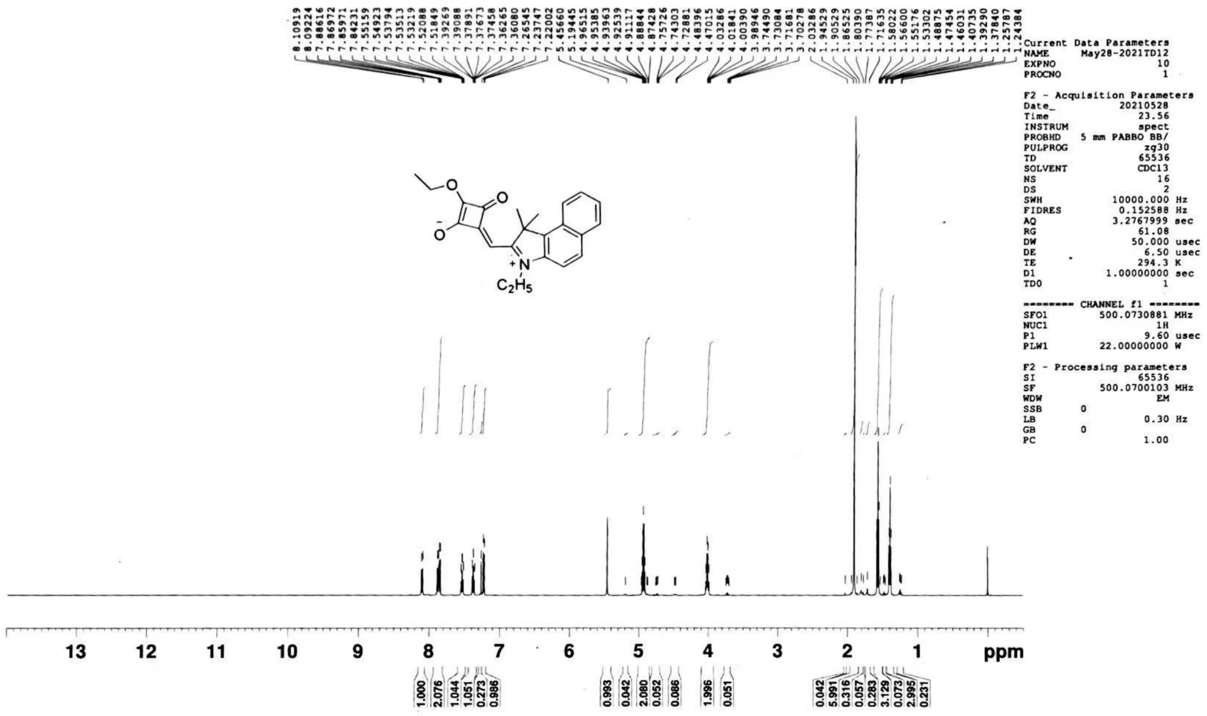

Figure S3. ^{1}H-NMR spectrum of **4** in $CDCl_3$.

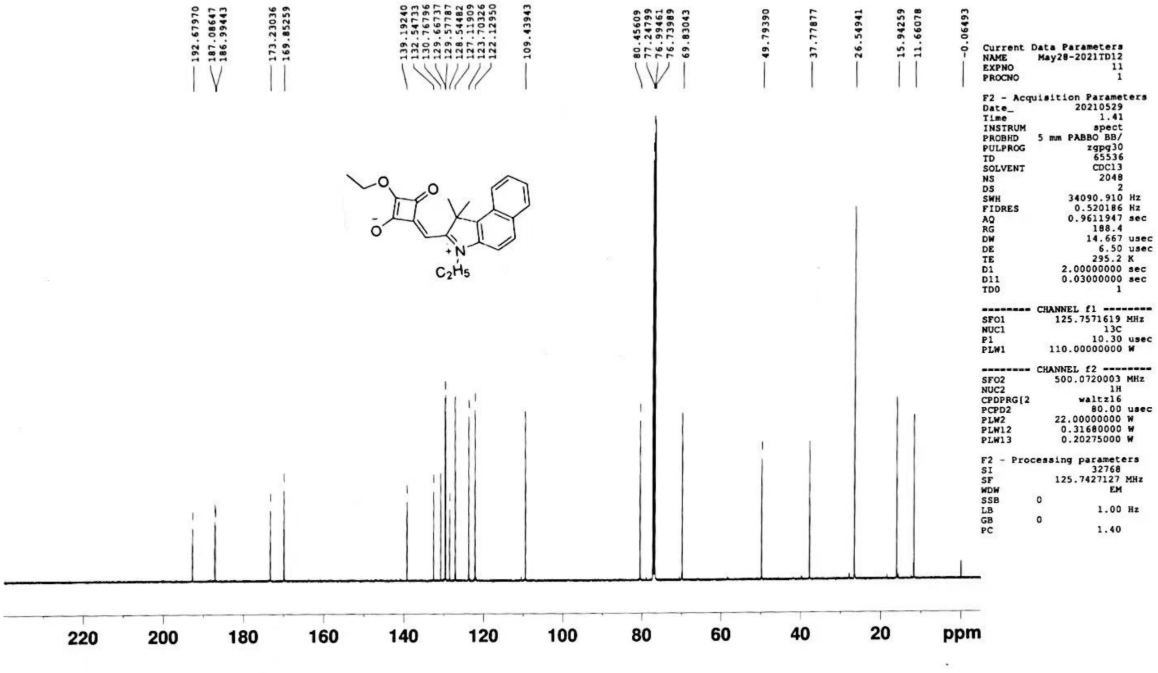

Figure S4. ^{13}C-NMR spectrum of **4** in $CDCl_3$.

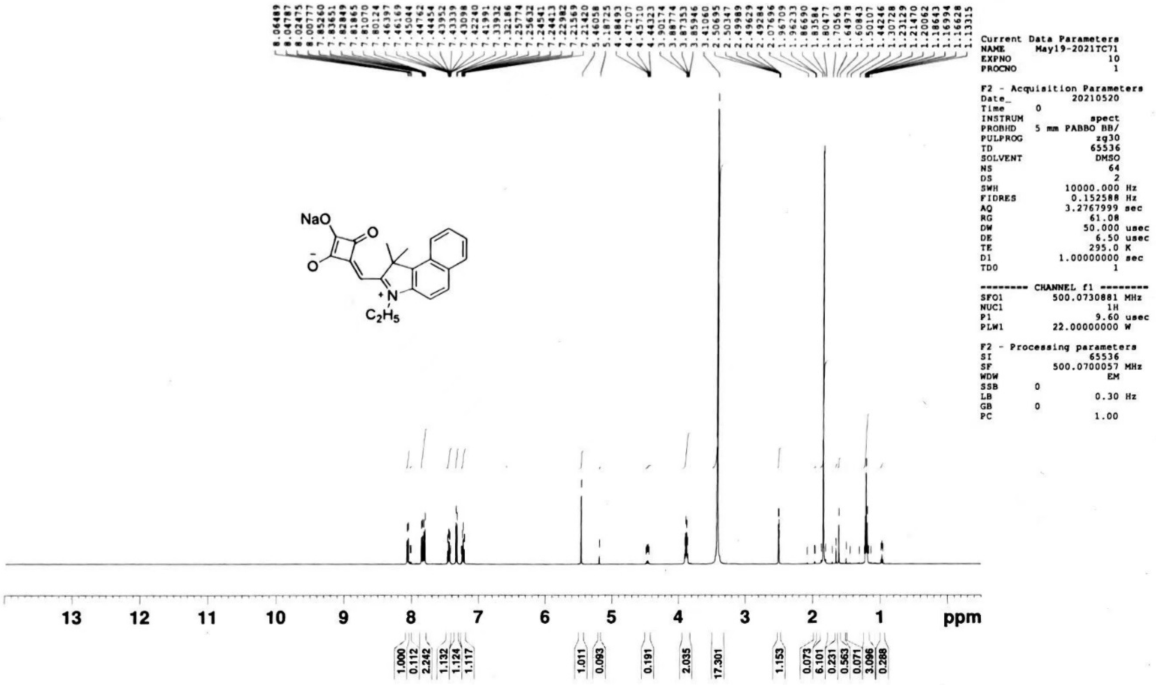

Figure S5. ^{1}H-NMR spectrum of **5** in DMSO.

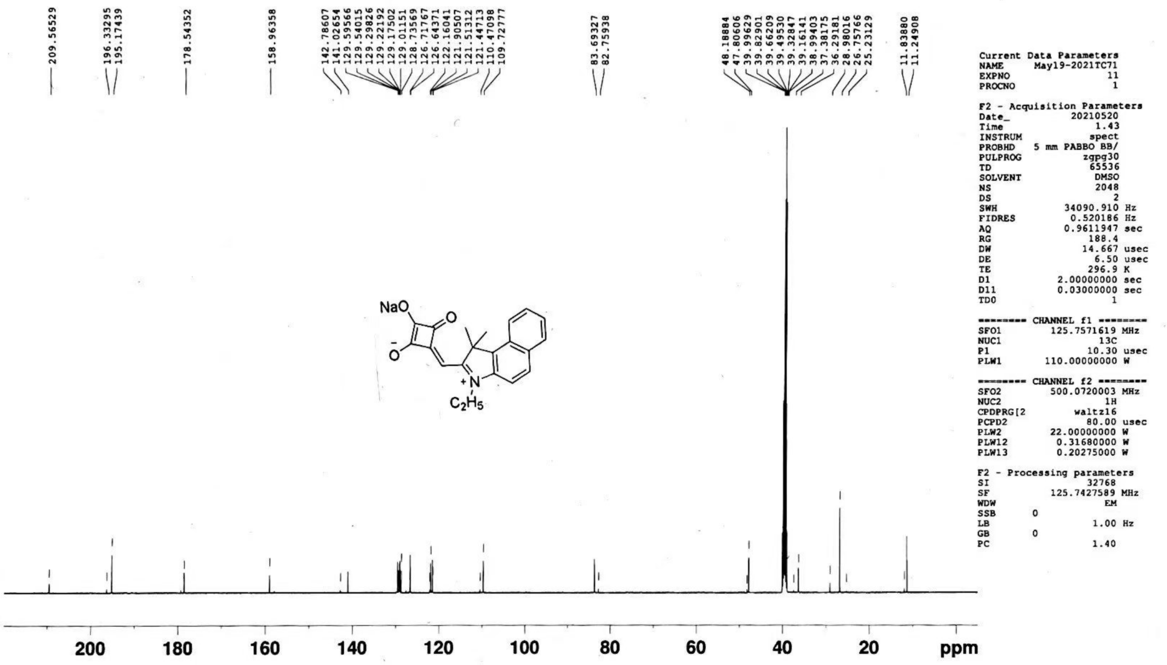

Figure S6. ^{13}C-NMR spectrum of **5** in DMSO.

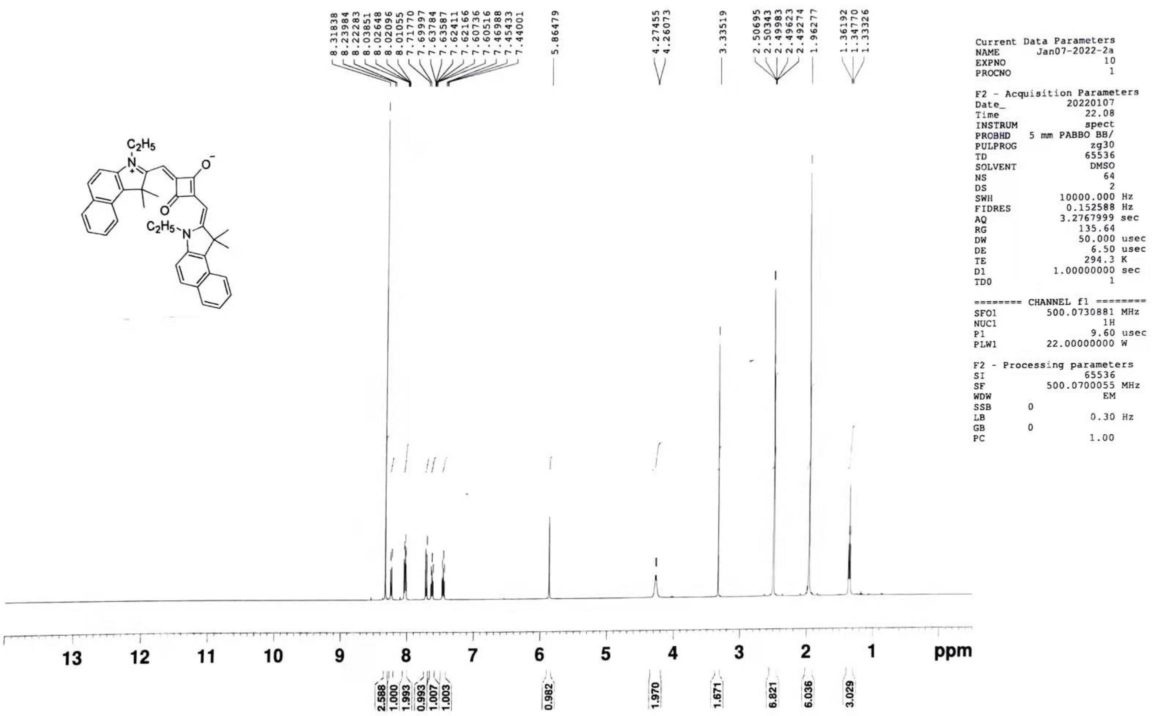

Figure S7. ^{1}H-NMR spectrum of **6** in DMSO.

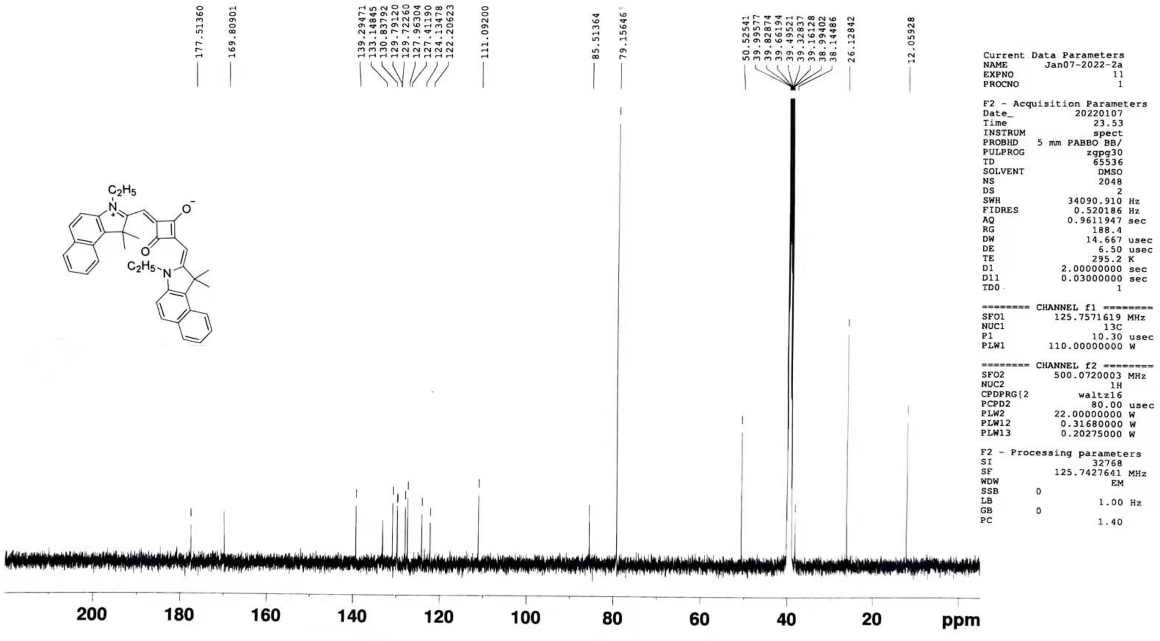

Figure S8. ^{13}C-NMR spectrum of **6** in DMSO.

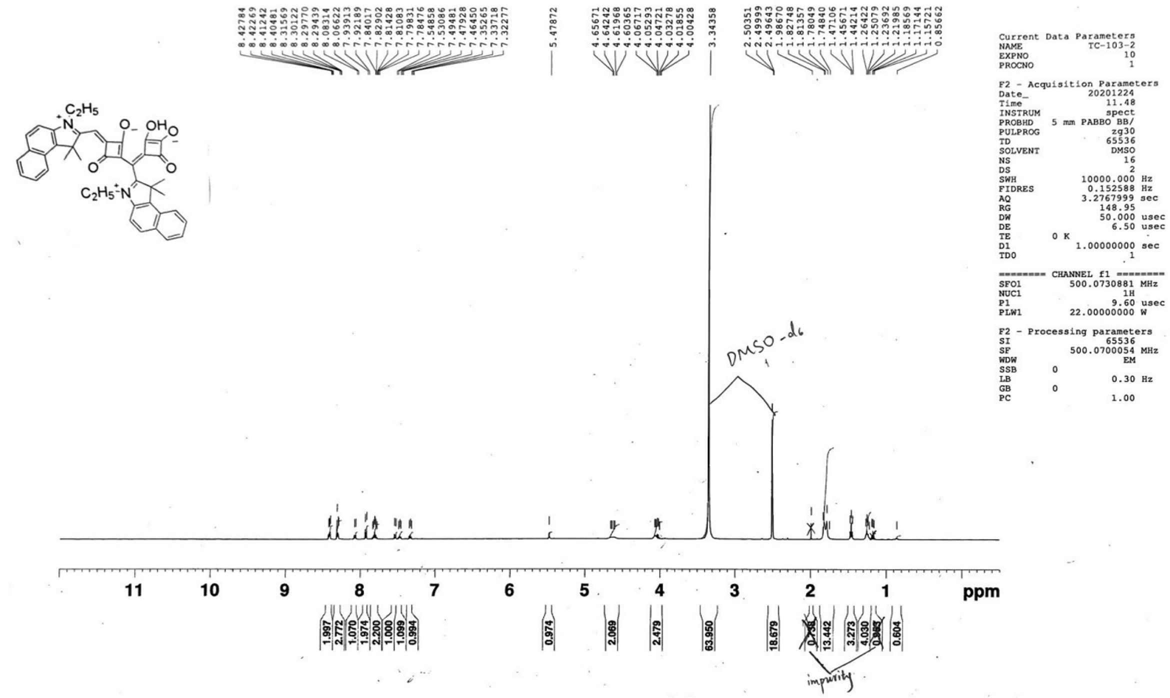

Figure S9. ^{1}H-NMR spectrum of **1** in DMSO.

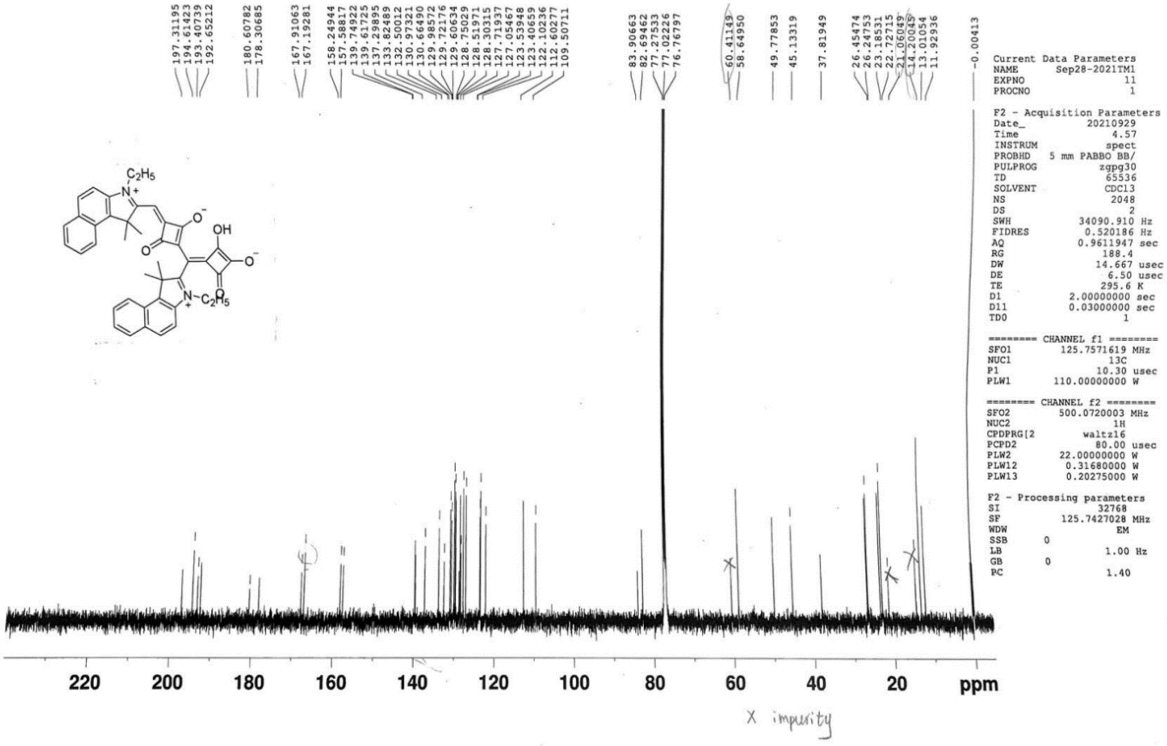

Figure S10. ^{13}C-NMR spectrum of **1** in $CDCl_3$.

2. Mass spectral analysis:

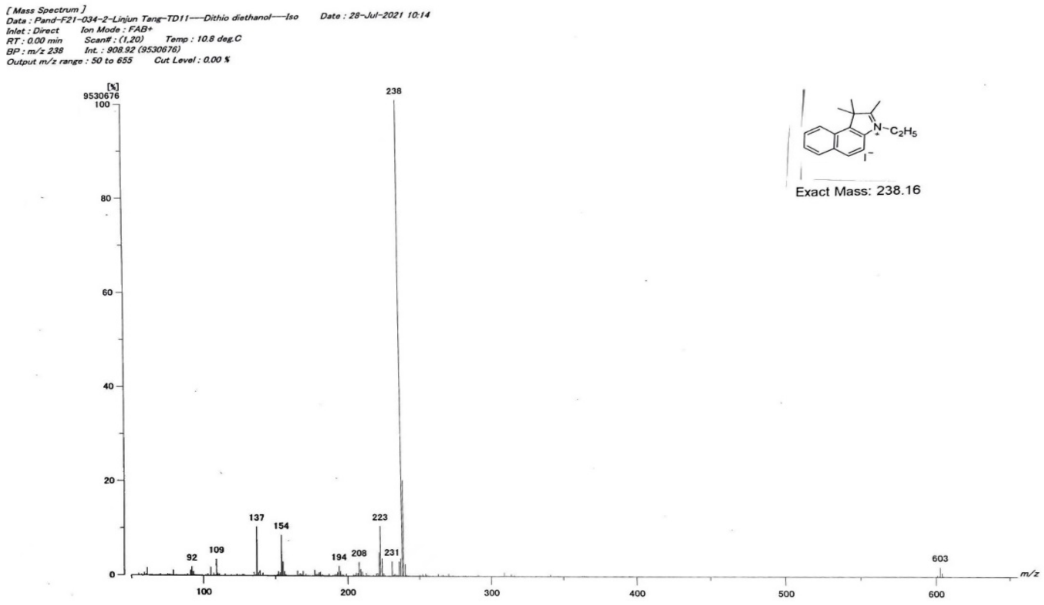

Data : Pand-F21-035-2-Linjun Tang-TD11==HR---Dithio diethanol---Iso   Date : 28-Jul-2021 11:12
Sample : -
Note : -
RT : 3.05 min   Scan# : 25
Elements : C 17/0, H 20/0, N 1/0
Mass Tolerance : 1000ppm, 5mmu if m/z < 5, 50mmu if m/z > 50
Unsaturation (U.S.) : -0.5 - 10.0

	Observed m/z	Int%	Err[ppm / mmu]	U.S.	Composition
1	238.1597	100.00	+0.5 / +0.1	8.5	C17 H20 N

Figure S11. HR-MS spectrum of **3**.

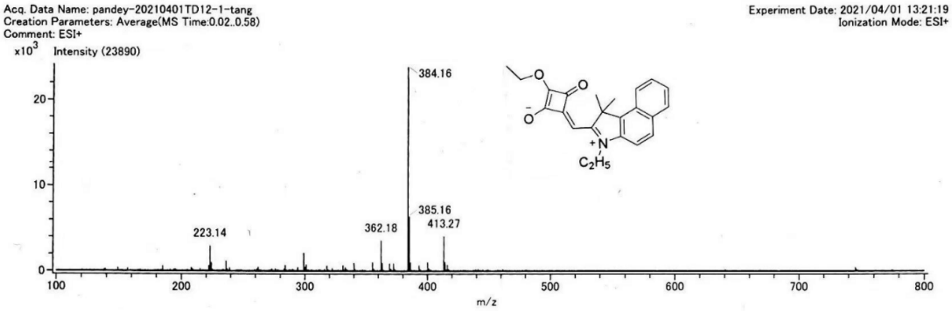

Peak#	m/z	Area	Area[% Max.]	Height	Height[% Max.]	Description
1	223.14	6136.41	10.96	2944.73	12.33	
2	224.14	2114.75	3.78	999.85	4.19	
3	236.14	2343.31	4.18	1196.35	5.01	
4	299.13	5114.78	9.13	2133.56	8.93	
5	340.10	2277.29	4.07	939.29	3.93	
6	355.12	2199.32	3.93	1038.76	4.35	
7	362.18	7506.31	13.40	3594.14	15.04	
8	363.18	2315.13	4.13	960.47	4.02	
9	369.14	2217.76	3.96	906.88	3.80	
10	372.39	2032.07	3.63	903.38	3.78	
11	384.16	56009.18	100.00	23890.36	100.00	
12	385.16	14121.54	25.21	6418.92	26.87	
13	386.17	2474.89	4.42	1006.68	4.21	
14	400.13	2713.99	4.85	1021.08	4.27	
15	413.27	9468.49	16.91	4096.11	17.15	
16	414.27	2589.94	4.62	1067.27	4.47	

Figure S12. TOF-MS spectrum of **4**.

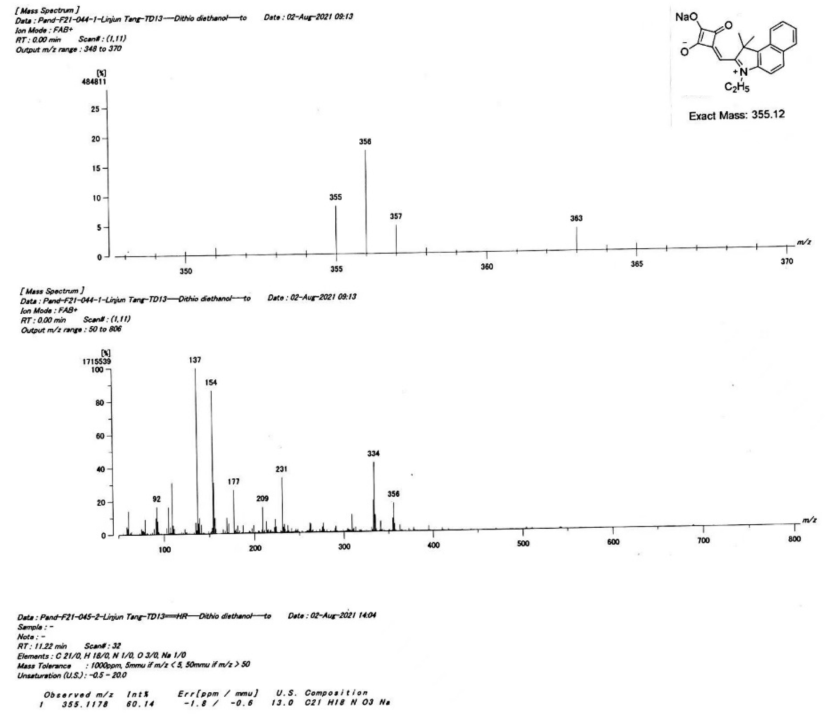

Figure S13. HR-MS spectrum of **5**.

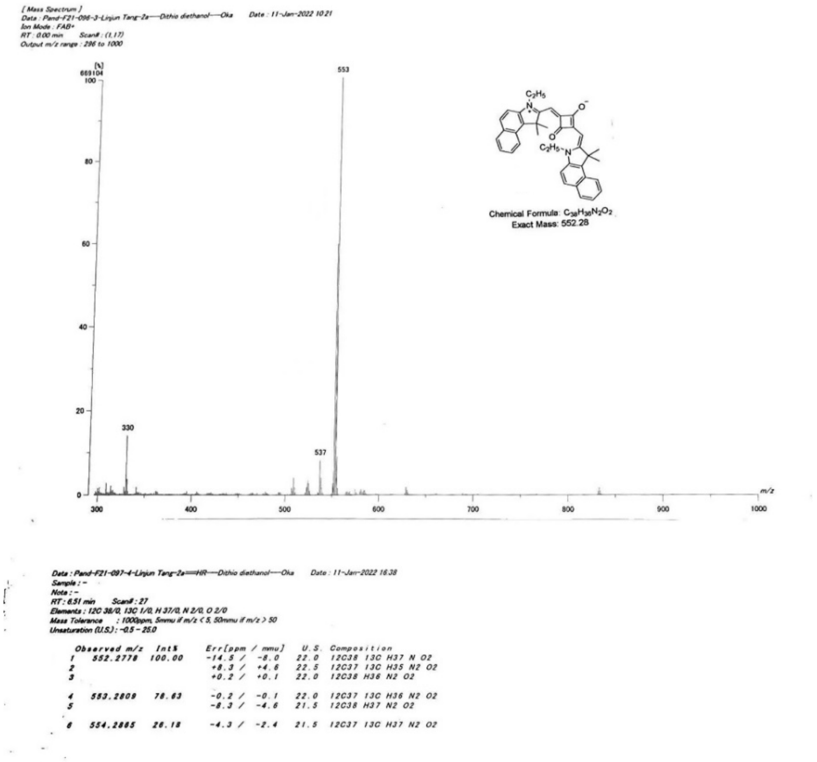

Figure S14. HR-MS spectrum of **6**.

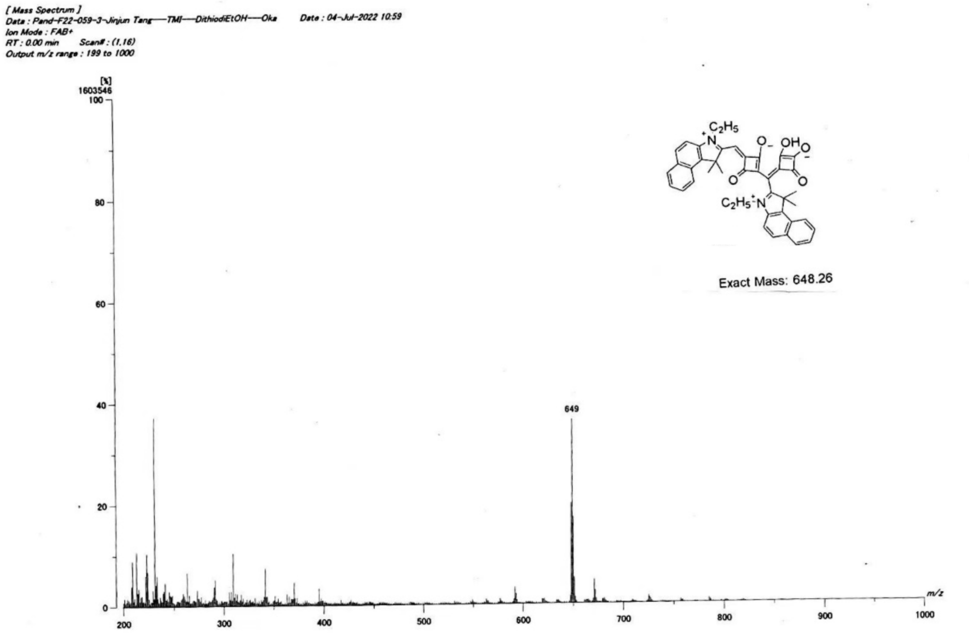

Data : Pand-F22-060-2-Jinjun Tang—TMI-==HR—DithiodiEtOH—Oka   Date : 04-Jul-2022 11:25
Sample : -
Note : -
RT : 1.72 min   Scan# : 8
Elements : 12C 42/0, 13C 2/0, H 37/0, N 2/2, O 5/5
Mass Tolerance : 1000ppm, 5mmu if m/z < 5, 50mmu if m/z > 50
Unsaturation (U.S.) : -0.5 - 30.0

	Observed m/z	Int%	Err[ppm / mmu]	U.S.	Composition
1	648.2639	64.93	+16.1 / +10.4	27.0	12C40 13C2 H34 N2 O5
2			+9.2 / +5.9	26.5	12C41 13C H35 N2 O5
3			+2.3 / +1.5	26.0	12C42 H36 N2 O5
4	649.2712	100.00	+15.2 / +9.9	26.5	12C40 13C2 H35 N2 O5
5			+8.4 / +5.4	26.0	12C41 13C H36 N2 O5
6			+1.5 / +1.0	25.5	12C42 H37 N2 O5
7	650.2757	44.24	+10.1 / +6.6	26.0	12C40 13C2 H36 N2 O5
8			+3.2 / +2.1	25.5	12C41 13C H37 N2 O5
9	651.2834	.12.68	+9.9 / +6.4	25.5	12C40 13C2 H37 N2 O5

Figure S15. HR-MS spectrum of **1**.

3. UV-Vis Spectroscopic Studies:

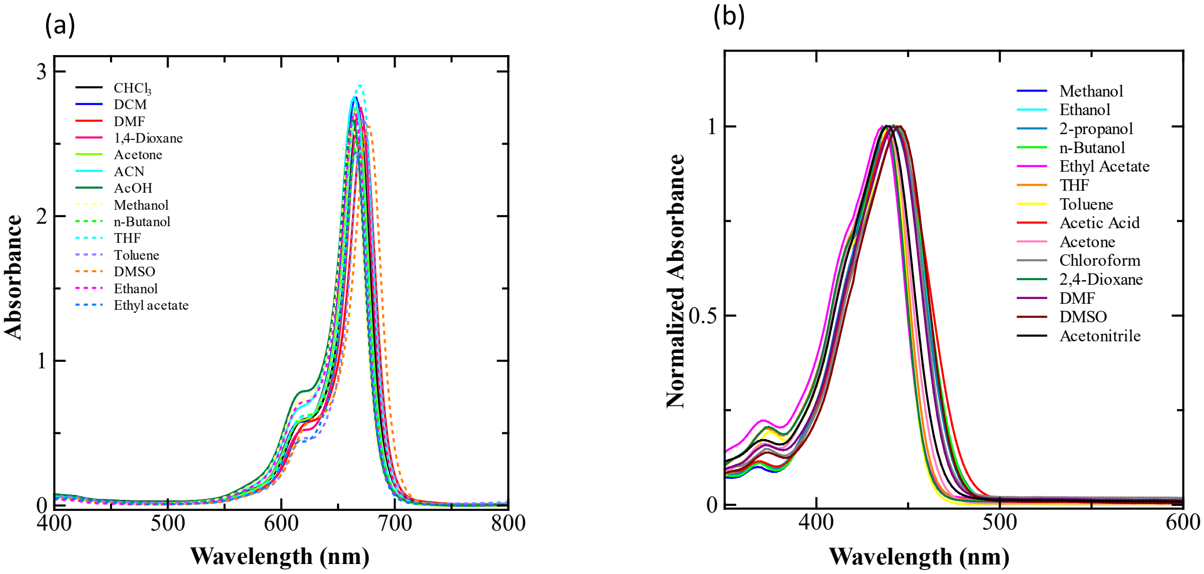

Figure S16. (a) UV-Vis spectra of **6** dissolved in different super dehydrated solvent (25 μM) and **(b)** UV-Vis spectra of **4** dissolved in different super dehydrated solvents (10 μM).

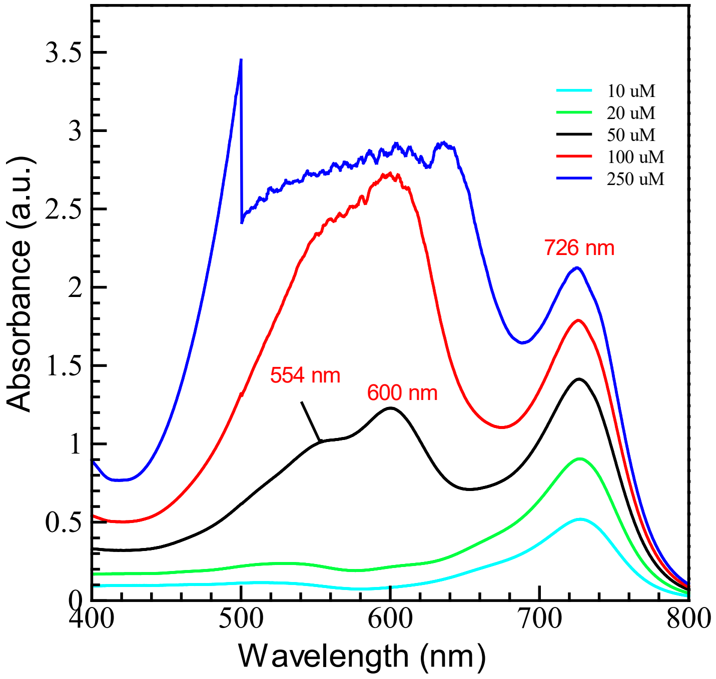

Figure S17. UV-Vis absorption of different concentration of DSQ **(1)** dissolved in DMF

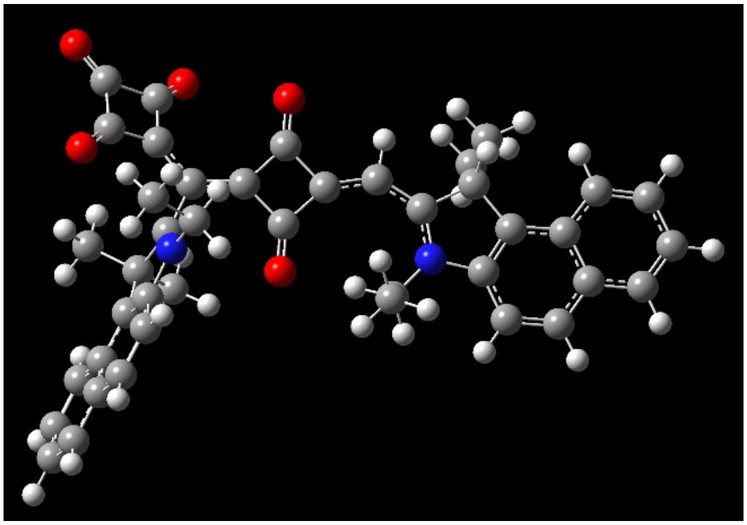

Figure S18. The three-dimensional structure of **1** utilizing Theoretical MO calculation (Gaussian G09 program)

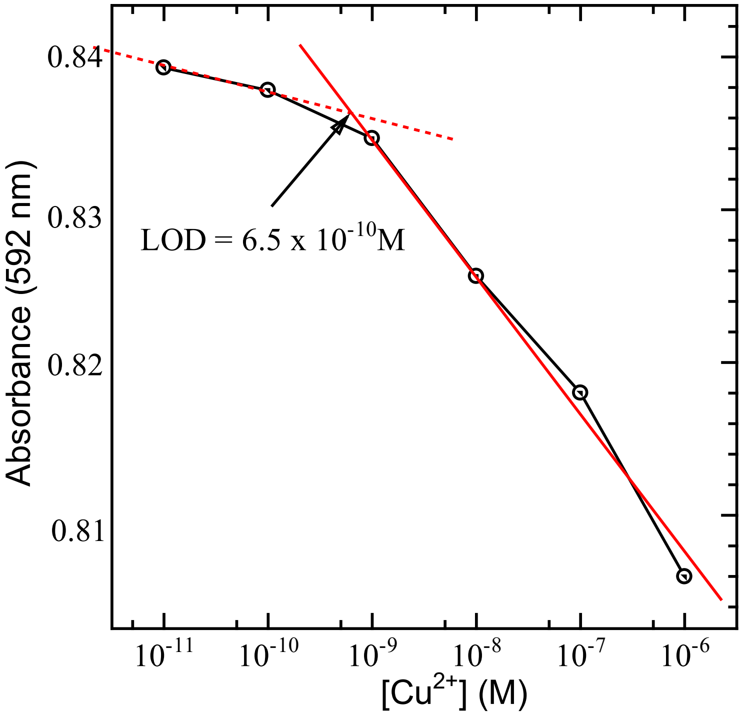

Figure S19. Change in absorption at 592 nm upon addition of Cu^{2+} ions showing limit of detection

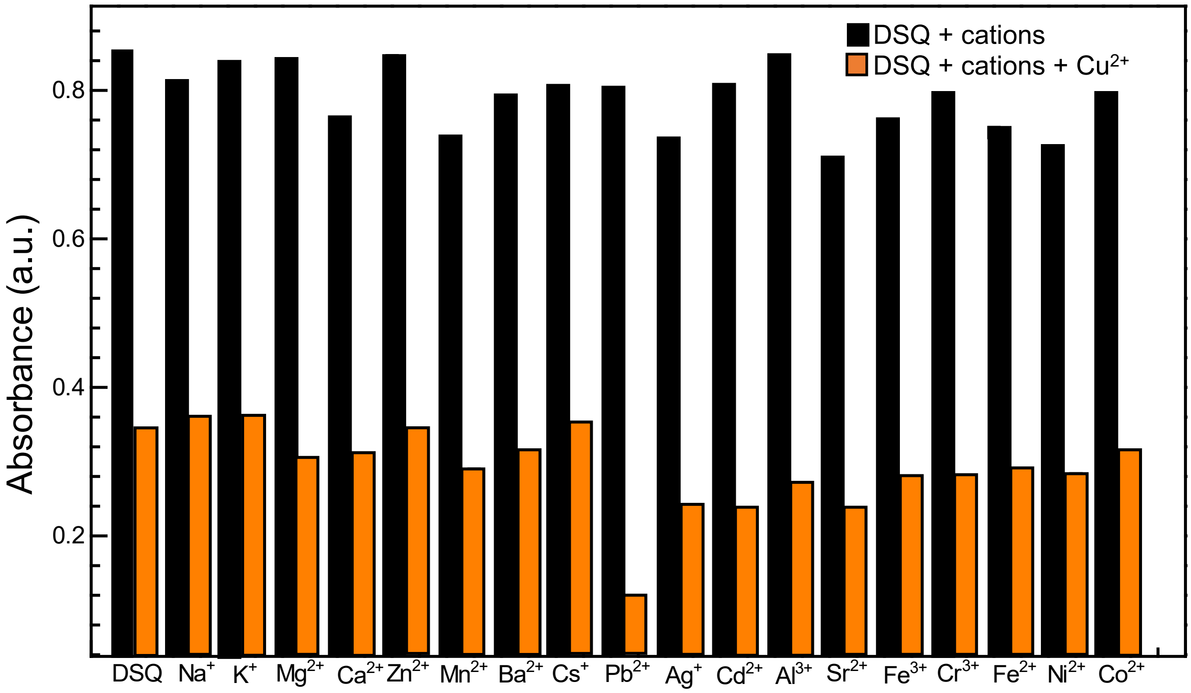

Figure S20. UV-Vis absorption at 592 nm of 10 μM DSQ **(1)** (dissolved in DMF/ACN, 1:99, *v/v*) in different ion solutions after addition of Cu^{2+}
